# A new species of *Cyanea* (Campanulaceae, Lobelioideae), from the Ko‘olau Mountains of O‘ahu, Hawaiian Islands

**DOI:** 10.3897/phytokeys.46.8694

**Published:** 2015-02-05

**Authors:** Margaret J. Sporck-Koehler, Tobias B. Koehler, Sebastian N. Marquez, Mashuri Waite, Adam M. Williams

**Affiliations:** 1 Hawai‘i Department of Land and Natural Resources, Division of Forestry and Wildlife, 1151 Punchbowl St. Rm. 325, Honolulu, HI 96813 USA; 2 Department of Botany, University of Hawai‘i at Mānoa, 3190 Maile Way, Honolulu, HI 96822, USA; 3 Papahana Kuaola 46-403 Haiku Road, Kaneohe, Hawai‘i 96744 USA; 4 Koehler Enterprises LLC., Honolulu, Hawai‘i 96813 USA; 5 Ko‘olau Mountains Watershed Partnership, 2551 Waimano Home Rd, Pearl City, HI 96782, USA

**Keywords:** Campanulaceae, conservation, *Cyanea*, endemic, Hawai‘i, Hawaiian Islands, IUCN Red List, Kōnāhua-nui, Ko‘olau Mountains, Lobeliads, Lobelioideae, O‘ahu, rare plants, *Rollandia*

## Abstract

*Cyanea konahuanuiensis* Sporck-Koehler, M. Waite, A.M. Williams, **sp. nov.**, a recently documented, narrowly endemic species from the Hawaiian Island of O‘ahu, is described and illustrated with photographs from the field. The closest likely relatives to the species, current conservation needs, and management future are discussed. It is currently known from 20 mature plants from two subpopulations and is restricted to a drainage below the Kōnāhua-nui summit (K1), the highest summit of the Ko‘olau Mountains, located on Windward O‘ahu. It differs from all other *Cyanea* species by its combination of densely pubescent leaves, petioles, and flowers; sparsely pubescent to glabrous stems, long calyx lobes, and staminal column being adnate to the corolla.

## Introduction

The Campanulaceae is a large, diverse, and cosmopolitan plant family with representatives inhabiting a wide range of ecosystems including tropical, subtropical, temperate, and even frigid zones with exceptional diversification in South Africa and Hawai‘i (Lammers 2007). One of the most exceptional adaptive radiation events known in the family is the monophyletic lobeliod group in Hawai‘i (Givnish et al. 2008). The Hawaiian lobelioids are a group of woody eudicots comprised of six genera, *Brighamia* A. Gray, *Clermontia* Gaudich., *Cyanea* Gaudich., *Delissea* Gaudich., *Lobelia* L., and *Trematolobelia* A. Zahlbr. Together they account for roughly ten percent of Hawaiian angiosperm diversity (Wagner et al. 1999, 2005) with a total of 128 taxa, 78 of which are currently recognized as Threatened and Endangered (T&E; USFWS http://www.fws.gov/endangered/).The lobelioids in Hawai‘i represent the largest adaptive radiation from a single colonization event known from any plant group restricted to an oceanic island chain (Oppenheimer and Lorence 2012, Givnish et al. 2008). According to the most current treatment of the Hawaiian Lobelioids (Lammers 2007), *Cyanea* (including the merger of the genus *Rollandia*) is the most species-rich genus in the radiation, comprised of 79 currently recognized species (54 T&E). A possible explanation for the impressive speciation in this genus is that the fleshy fruits are poorly distributed by Hawai‘i’s native forest birds, which do not typically travel long distances, leading to parallel speciation events on multiple islands (Givnish 2008). *Cyanea* occurs in mesic to wet forests across the Hawaiian archipelago and includes many taxa with restricted distributions, most of which are single island endemics.

**Figure 1.**
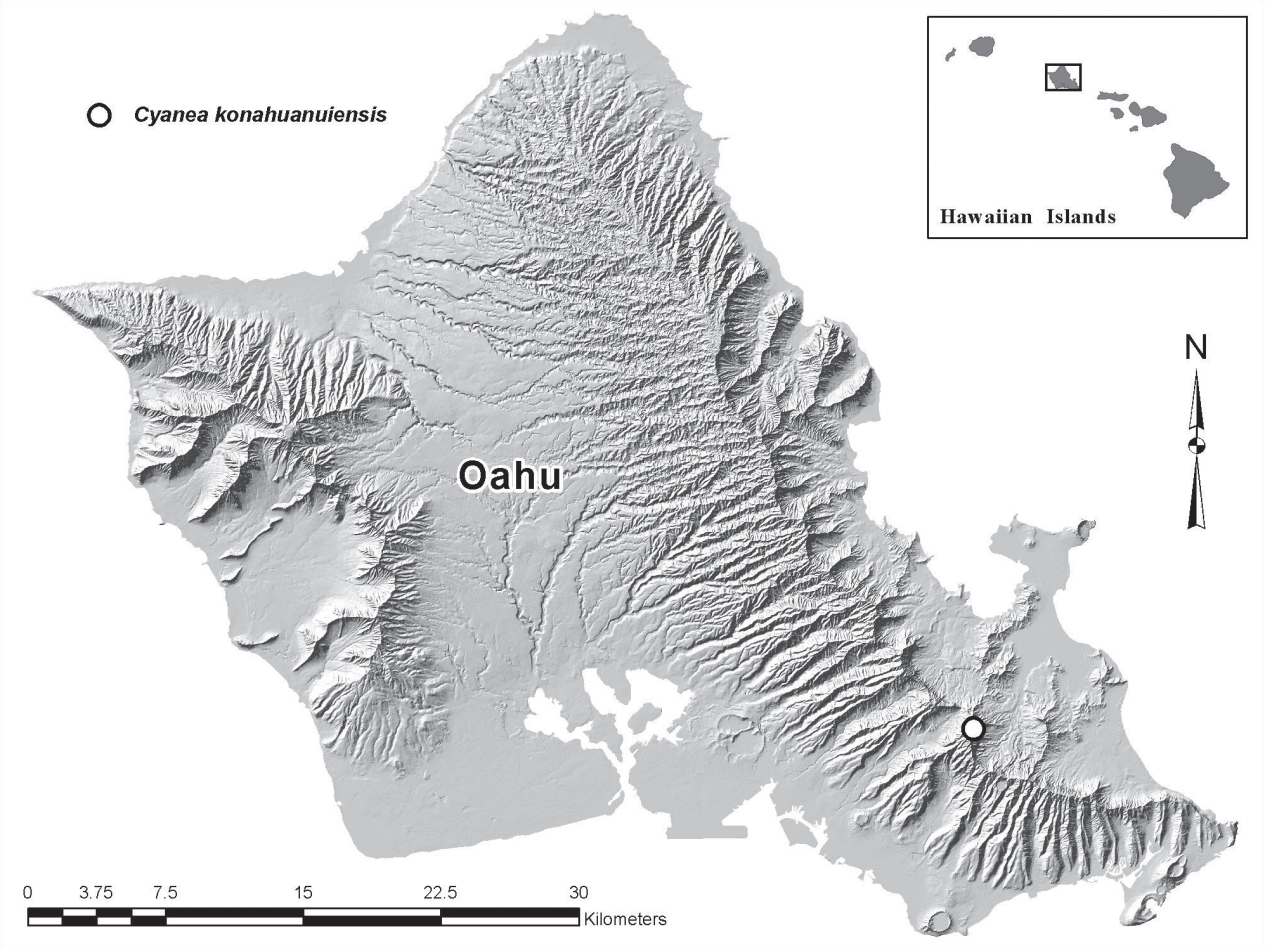
Distribution map of *Cyanea konahuanuiensis* showing site of population on South East O‘ahu in the Ko‘olau Mountain Range.

In September 2012, the Kōnāhua-nui summit area of the Ko‘olau Mountains on the island of O‘ahu was surveyed (Figure 1). The target species was *Cyanea humboldtiana* (Gaudich.) Lammers, Givnish & Sytsma, a species federally listed as Endangered, and endemic to the Ko‘olau Mountains. It was hoped to locate additional individuals to monitor and manage as a part of species recovery efforts. We summited K1 (the highest of the two peaks of Kōnāhua-nui) and descended into a stream drainage and once near the bottom of the gulch, directly adjacent to the stream, several plants were discovered of a *Cyanea* with hairy leaves and petioles, glabrous stems, and long, hairy calyx lobes. There were no flowers present on the plants and only one immature infructescence. The specific taxon could not be confirmed in the field and it was decided to take photos and collect a dropped and decaying leaf for further investigation.

Preliminary research in the following weeks consisted of examining the plants using photos and the one fallen, partially decayed leaf collected from the field. After reviewing the current treatments of *Cyanea*, sharing the photos with local experts, and looking at specimens at the Bishop Museum, it was concluded that the species in question was most likely undescribed. To be certain, fertile specimen was required.

**Figure 2.**
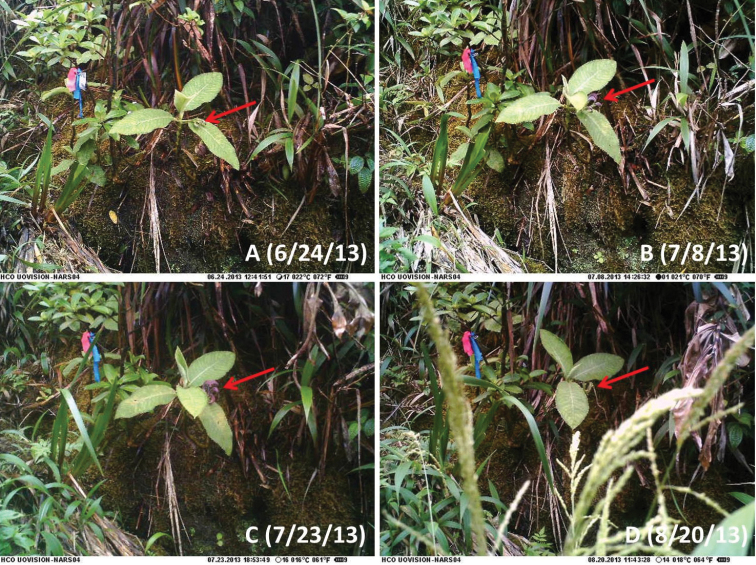
*Cyanea konahuanuiensis*. Game camera time lapse series illustrating flower and early fruit development over a nearly two month time frame. Panels **A–D** each illustrate a different stage of development. Red arrows point to developing flower and fruit features.

Thereafter, trips to the remote population were made every two to three months. These subsequent expeditions surveyed the surrounding area and revealed the presence of additional individuals. In June 2013, an automated game camera was installed connected to cellular phone service that transmitted a photo of one of the plants three times per 24 hour period (Figure 2). This camera allowed us to monitor the flower development and visit the plants again when we were certain the flowers would be fully mature.

In addition, type specimens of all of the Hawaiian Lobeliads were examined in the Herbarium Pacificum of the Bernice Pauahi Bishop Museum (BISH) collection, with special attention given to *Clermontia*, *Cyanea*, *Delissea*, and the formerly recognized generic group, *Rollandia* Gaudich. None of the Hawaiian Campanulaceae was a match to the *Cyanea* under study.

There are several species that share some characteristics with *Cyanea konahuanuiensis* including: *Cyanea crispa* Gaudich., *Cyanea calycina* Gaudich., *Cyanea humboldtiana* Gaudich., and *Cyanea pilosa* A. Gray (Table 1). For example, *Cyanea crispa*, *Cyanea calycina*, and *Cyanea humboldtiana* all share a staminal column that is adnate to the corolla tube, similar overall stature and leaf shape, presence of some leaf pubescence (though not to the same degree as *Cyanea konahuanuiensis*), and similar number of flowers in each inflorescence (*Cyanea konahuanuiensis* also shares the characteristic of pendant inflorescences but to a lesser degree than *Cyanea humboldtiana*). However, they all differ in the degree of leaf pubescence (*Cyanea konahuanuiensis* being significantly more hairy), calyx lobe length, and corolla surface characteristics.

**Table 1.** Selected traits for *Cyanea konahuanuiensis* compared to four *Cyanea* taxa sharing some similar morphological characteristics. Abbreviations: O = O‘ahu, H = Hawai‘i Island.

Species	Plant height (m)	Leaf length (cm)	Leaf width (cm)	Leaf shape	Adaxial leaf surface char.	Abaxial leaf surface char.	Peduncle Length (mm)	Number of flowers in inflorescence	Calyx lobe shape	Calyx lobe length (mm)	Hypanthium length (mm)	Flower Color	Corolla surface char.	Island found on
*Cyanea konahuanuiensis*	0.57–0.69	20–33	10–16	elliptic–oblong	densely hirsute, juveniles subtlety muricate	densely hirsute, juveniles subtlety muricate	50–122	3–12	linear to linear-oblong	16–18	10–15	dark magenta/ purple w/ some lighter streaking as flowers age	densely pubescent	O
*Cyanea calycina*	1–3	15–60	5.5–14	elliptic–oblanceolate	glabrous, juveniles muricate	densely pubescent w/branched & clustered hairs	20–100	4–16	oblong- ovate	4–10	6–12	pale–dark magenta, rarely pale greenish w/ lighter or darker longitudinal stripes	pubescent	O
*Cyanea crispa*	0.3–1.3	30–75	9–16	broadly obovate	glabrous	glabrous or pubescent	20–30	3–8	ovate–oblong	6–12	8–12	pale magenta w/ darker longitudinal stripes	pubescent	O
*Cyanea humboldtiana*	1–2	18–45	7–16	obovate–broadly elliptic	glabrous	pubescent	80–250	5–12	oblong	4–10	8–12	dark magenta, rarely white	pubescent	O
*Cyanea pilosa*	0.8–2	15–42	8–15	broadly elliptic–broadly obovate	pubescent	whitish green, densely pubescent	15–110	6–28	narrowly triangular	2–5	4–6	white	pubescent	H

**Figure 3.**
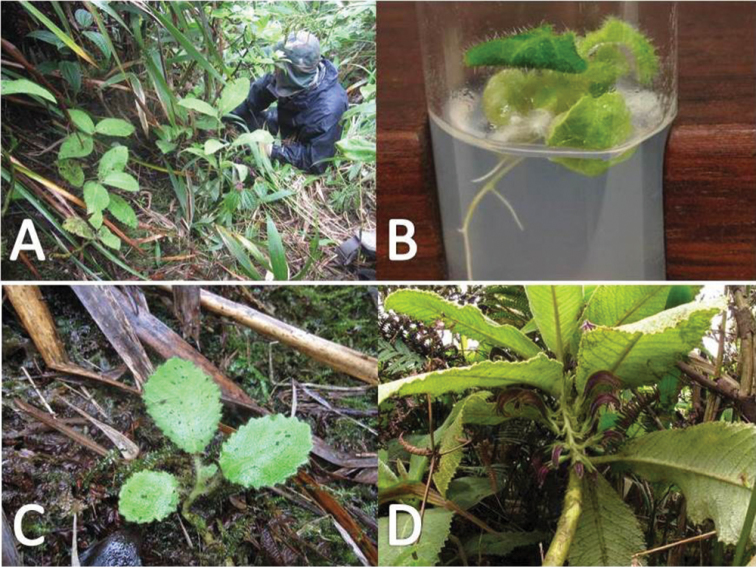
*Cyanea konahuanuiensis*. **A** habit, photo with Adam M. Williams crouched down next to plant for scale **B** Seedlings growing in test tube in micropropagation lab **C** juvenile plant in the field **D** flowering stem with pendent inflorescence captured (photos by Margaret J. Sporck-Koehler ).

**Figure 4.**
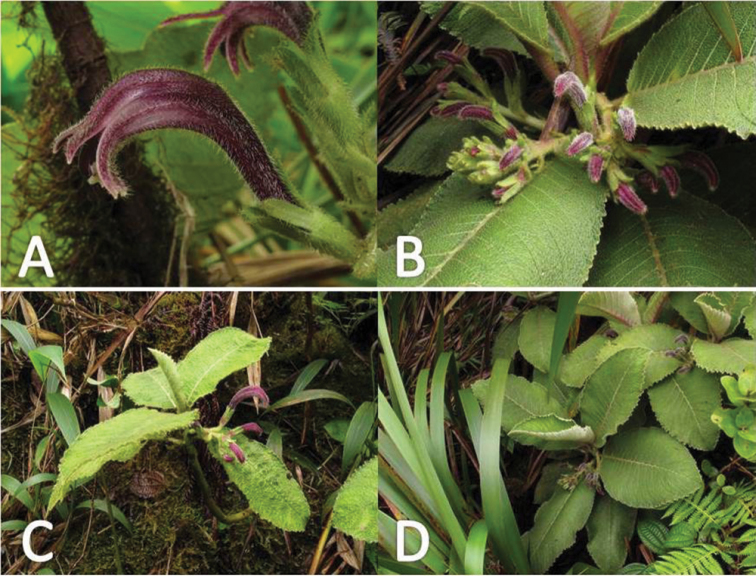
*Cyanea konahuanuiensis*. **A** detail of corolla tube in profile. Dense pubescence and long calyx lobes apparent **B** close up view of buds **C** view of plant habit depicting single-stalked individual **D** view of plant habit depicting multi-stalked individual (photos by Tobias B. Koehler).

**Figure 5.**
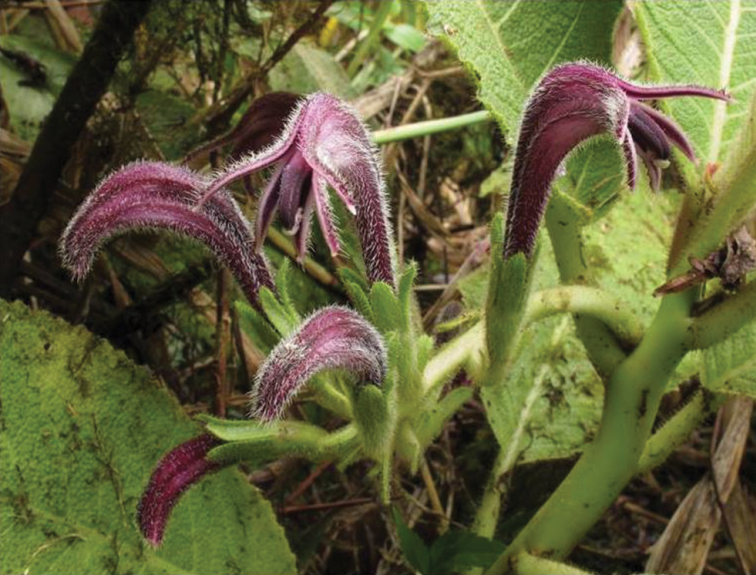
*Cyanea konahuanuiensis*. Inflorescence/flowers illustrating pendant inflorescence, dense pubescence of flowers, and long calyx lobes. Pubescent petioles and hairless stems are also apparent (photo by Tobias B. Koehler).

**Figure 6.**
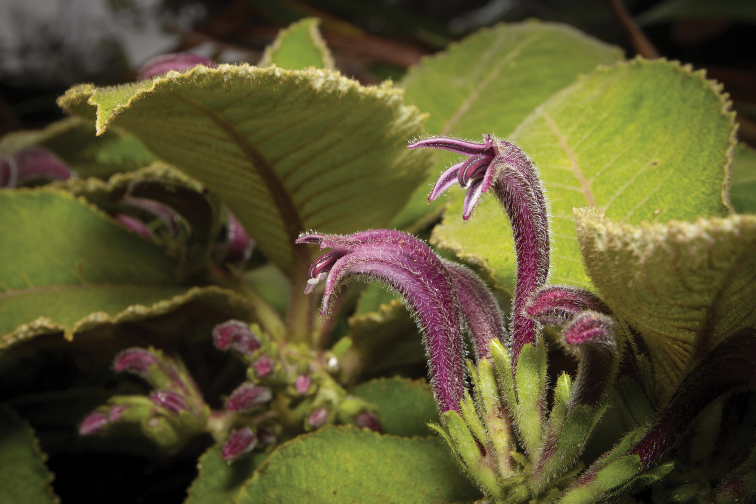
*Cyanea konahuanuiensis*. Inflorescence/flowers illustrating dense pubescence of flowers, long calyx lobes. Pubescent leaves also apparent (photo by Chris A. Johns).

**Figure 7.**
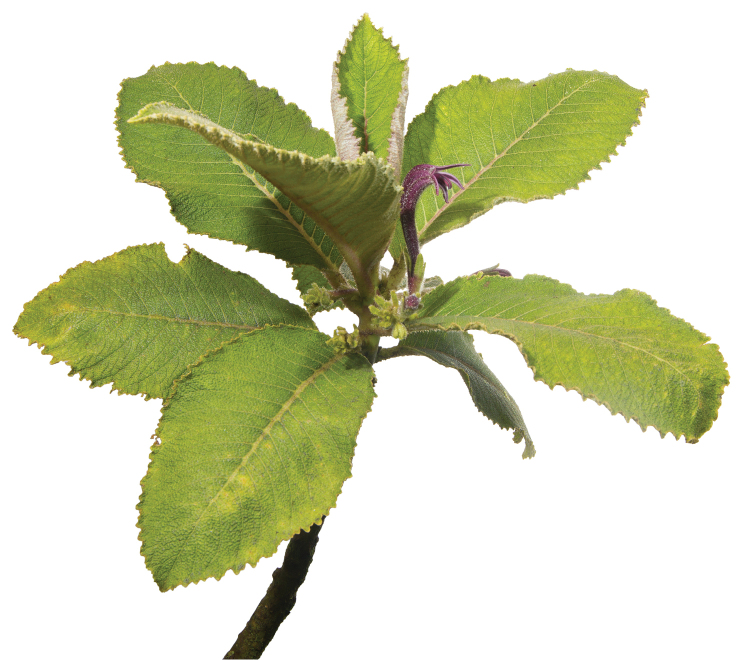
*Cyanea konahuanuiensis*. Solitary stem illustrating typical leaf arrangement clustered distally near the stem terminus (photo by Chris A. Johns).

**Figure 8.**
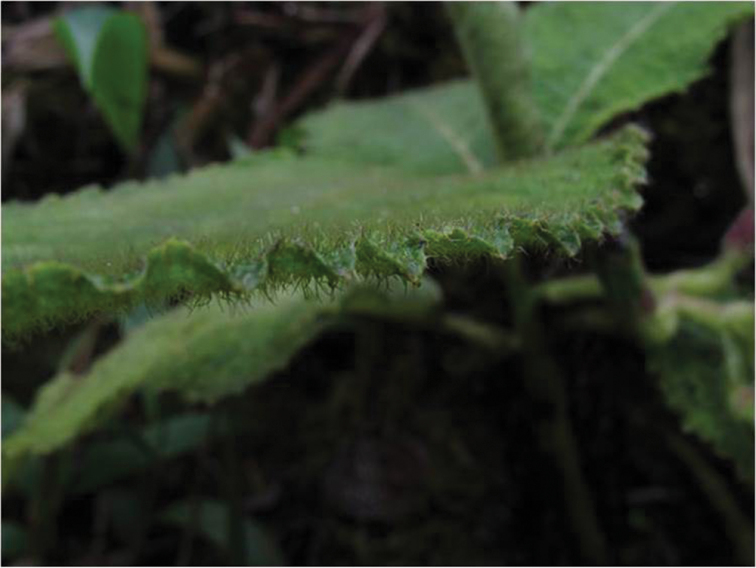
*Cyanea konahuanuiensis*. Photo of leaf illustrating dense pubescence on both adaxial and abaxial leaf surfaces (photo by Tobias B. Koehler).

The Hawai‘i Island endemic *Cyanea pilosa* shares a comparable leaf shape and is similarly pubescent (though dissection scope inspection revealed that hair structure differs) to *Cyanea konahuanuiensis*, but the latter lacks hairy stems and has more abundant hair on both leaf surfaces. Also, flower color and calyx lobe length differ radically, *Cyanea pilosa* usually having white flowers and much shorter calyx lobes (2–5 mm long) and *Cyanea konahuanuiensis* having brilliant purple flowers. Lastly, unlike *Cyanea konahuanuiensis*, the staminal column of *Cyanea pilosa* is not adnate to the corolla tube (Figures 3, 4, 5, 6, 7, 8 and Table 1).

Because so few individuals of *Cyanea konahuanuiensis* are known, collections were limited to two fertile vouchers, each including one inflorescence and two leaves. Flowers were also collected from three individual plants and preserved in alcohol for precise floral analysis (see Specimens Examined section). In order to ensure limited damage to the population and to further conserve the species, detailed measurements were taken of five, reproductively mature and flowering, live plants in the wild for most of the descriptive information in this paper. For corolla tube lobe length and width, surface characteristics, and anther length and surface characteristic data from the preserved flowers were utilized. Seed data are taken from living material deposited at the University of Hawai‘i Harold L. Lyon Arboretum micropropagation facility. Selected measurements from the dried holotype specimen are included parenthetically in the description.

## Systematics

### Cyanea konahuanuiensis

Taxon classificationPlantaeAsteralesCampanulaceae

Sporck-Koehler, M. Waite & A.M. Williams sp. nov.

urn:lsid:ipni.org:names:77145079-1

Figures 3 , 4 , 5 , 6 , 7

#### Note.

Species believed to be allied to *Cyanea humboldtiana* (Gaudich.) Lammers, Givnish & Sytsma, but primarily differs in its longer calyx lobes (16–18 mm long); and its more densely pubescent leaves, petioles, flowers, and differing flowering period. *Cyanea humboldtiana* has leaves, petioles, and flowers that are more sparsely pubescent with shorter trichomes; leaf margins are callose-crenulate, floral bracts have acute apices, and its calyx lobes are ovate, acuminate and considerably shorter (4–10 mm long). The two species are reproductively isolated from each other due to lack of overlapping flowering periods (*Cyanea konahuanuiensis* flowers from June–August and *Cyanea humboldtiana* from October–December), and they are not known to intergrade.

#### Type.

**Figure 9.**
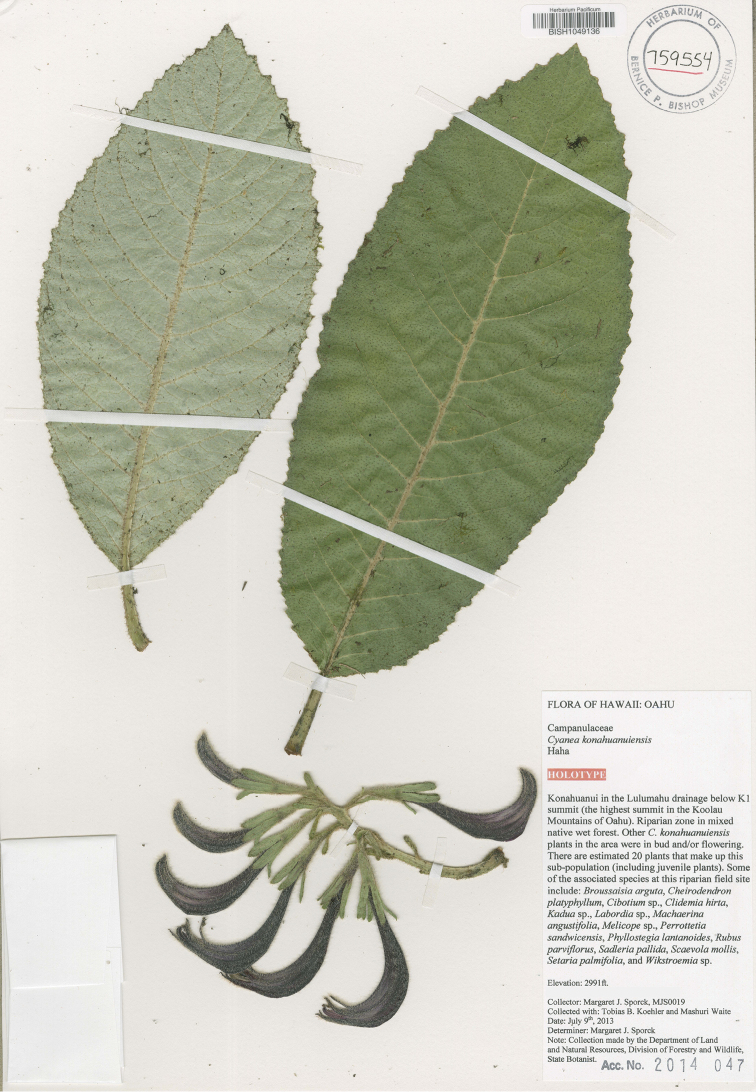
*Cyanea konahuanuiensis*. Photo of holotype (photo courtesy of Bishop Museum).

USA, Hawai‘i, O‘ahu Island, Ko‘olau Range: Kōnāhua-nui, near summit, 912m (2991 ft), 9 July 2013, M. Sporck, T. Koehler, & M. Waite MJS 0019 (holotype: BISH 1049136), (Figure 9).

#### Description.

*Unarmed shrubs* 57–69 cm high, with 1–6 stems originating at the base; stems light green and darkening to a light brownish gray closer to the base, erect to decumbent, 58–119 cm in length, some branches partially resting on the ground, occasionally rooting when in contact with the soil or moss producing aerial roots, leaf scars subcircular, 9.5–12 × 6.5–11.8 mm, upper end of leaf scar depressed, basal portion slightly raised; leaf scar with a protuberance; latex white. *Leaves* clustered distally near end of stems, petiolate; petioles 2–4.2 cm long, pubescent; blades elliptic to oblong, in adult plants 20–33 × 10–16 cm, base cuneate to rounded, occasionally slightly truncate, apex acute to sub-obtuse, margins serrate to serrate-dentate, dull grayish-green on adaxial surface and dull greenish white on abaxial surface, stiff, slightly fleshy, both surfaces densely hirsute and minutely muricate; in juvenile plants leaves are less stiff, margins dentate, and hairs softer. *Inflorescences* axillary just above the leaf, up to 4 per stem, young inflorescences roughly perpendicular to stem, larger and more developed inflorescences pendant, 3–12 flowered, peduncles 5–12.2 cm long (dried specimen 7.7 cm long), pubescent. *Flowers* on pedicels 7–14 mm long (5–10 mm when dried), pubescent, subtended by linear bracts 7–18 × 2–6 mm, apex obtuse, margins entire, densely pubescent; hypanthium 10–15 mm (9–14 mm when dried) × 7–10 mm (5–7 mm when dried), obovoid to cylindrical, pubescent; calyx lobes linear to linear-oblong,16–18 × 5–7 mm (13–17 × 3–5 mm when dried), apex acute to subobtuse, retained on immature (green) fruits (no mature fruit seen); corolla dark purple with some lighter streaks developing with age, tubular, laterally compressed, curved, 86–99 mm long (80–95 mm when dried) × 12–13 mm wide medially (9–12 mm when dried), externally densely pubescent, internally glabrous, the lobes linear-subulate, 10–16 mm long × 5 mm wide at the base, reflexed, c. 1/4–1/3 as long as the tube; staminal column glabrous, adnate to corolla for half its length, anthers 9–10 mm long, scantily pubescent, the lower two with apical tufts of white hairs 2–3 mm long. *Fruits* berries (mature fruits not seen), immature fruits densely pubescent, with calyx lobes persistent. *Seeds* from immature fruits numerous, embedded in green pulp, obovoid, 0.74–0.84 × 0.58–0.64 mm, testa medium to dark brown, shiny and smooth.

#### Distribution.

Known only from the Kōnāhua-nui summit area in the Ko‘olau Mountains of O‘ahu, Hawaiian Islands. The population is on land owned by the State of Hawai‘i, Department of Land and Natural Resources (DLNR), and is part of the Honolulu Watershed Forest Reserve (Figure 1).

#### Habitat and ecology.

*Cyanea konahuanuiensis* occurs in wet forest sites at elevations from 884 to 932 m. The associated native Hawaiian plant species include *Broussaisia arguta* Gaudich., *Cheirodendron platyphylla* (Hook & Arn.) Seem., Dubautia laxa (Hook. & Arn.) *Machaerina angustifolia* (Gaudich.) T. Koyama, *Metrosideros rugosa* A. Gray, *Perrotettia sandwicensis* A. Gray, *Phyllostegia grandiflora* (Gaudich.) Benth., *Polyscias gymnocarpa* (Hillebr.) Lowry & G. M. Plunkett, *Sadleria pallida* Hook. & Arn., *Scaevola mollis* Hook. & Arn., and species of *Cibotium* Kaulf., *Kadua* Cham. & Schltdl., *Labordia* Gaud., *Melicope* J.R. Forst. & G. Forst., and *Wikstroemia* Endl. Introduced naturalized plant species include: *Ageratina adenophora* (Spreng.) R.M. King & H. Rob., *Clidemia hirta* (L.) D. Don, *Hedychium gardnerianum* Sheppard ex Ker Gawl., *Rubus rosifolius* Sm., and *Setaria palmifolia* (J. König) Stapf. Bryophyte species are prevalent and include *Distichophyllum freycinetii* (Schwägr.) Mitt., *Plagiochila diflexa* Mont. & Gottsche, and species of *Bazzania* S. Gray. The native arthropod, *Megalagrion oahuense* Blackburn (a Hawaiian endemic damselfly) has been observed on *Cyanea konahuanuiensis*. Soil is of basaltic origin and typical of wet forest sites on O‘ahu and the average annual rainfall is approximately 2600 mm (Giambelluca et al. 2013). The plants occupy a gulch both at the base and middle of relatively steep slopes which results in direct sunlight exposure occurring for a few hours close to midday and varying seasonally. Plants occur on both northwest and south facing slopes of the gulch, mostly along the banks of intermittent streams, but can also be found several meters from the stream with no apparent preference. Small stem fragments that are detached from plant have been observed to take root, forming new clones.

#### Phenology.

*Cyanea konahuanuiensis* has been observed flowering from June–August with fruit developing from August–October. All observed fruits have aborted, been eaten, or decomposed before maturity. The lifespan and time to maturity of the species is unknown. Immature, nearly aborted fruits have been collected when all others had aborted. Fruits have been submitted to the Lyon Arboretum Micropropagation lab where some of the seeds have germinated and it is interesting to note that the seedlings have densely pubescent leaves (Figure 3).

#### Etymology.

The specific name pays homage to the twin-peaked (946 m and 960 m) Kōnāhua-nui Pu‘u (summit), the tallest peaks in the Ko‘olau Mountain range on windward (east) O‘ahu. *Lit.* Large fat innards (Pukui et al. 1974), + Latin suffix –*ensis*, indicating a place of origin or belonging. “In one story a giant threw his great testicles (*Kona hua nui*) at a woman who escaped him.” (Pukui et al. 1974). Kōnāhua-nui has significance not only because it is the highest peak in the Ko‘olau Range, but because the summit area is a largely intact native ecosystem in relative close proximity to Honolulu, the largest city in the State of Hawai‘i. To our knowledge, there has never before been a plant species named after this beautiful and biologically important locality. After seeking counsel with Hawaiian cultural practitioners Kaua Neumann and Kīhei Nahale-a (pers. comm. 2014), it is proposed to give the species a Hawaiian name of *Hāhā mili‘ohu*, meaning “The Cyanea that is caressed by the mist”.

#### Conservation.

*Cyanea konahuanuiensis* is a critically imperiled species (see Conservation status below) due to its low population numbers and exceptionally narrow endemism. Some of the conservation obstacles to overcome include probable loss of most, if not all, of its native avian pollinators and dispersers, and suspected herbivory by introduced taxa such as rats, terrestrial gastropod mollusks (slugs), and feral pigs (*Sus scrofa*). Invasive plant species are becoming increasingly common even in relatively hard to access sites along and near mountain summits in Hawai‘i. Species such as *Ageratina adenophora*, *Clidemia hirta*, *Erigeron karvinskianus*, *Hedychium gardnerianum*, *Rubus rosifolius* and *Setaria palmifolia* are competing with *Cyanea konahuanuiensis* and other native species for space and resources. It is conceivable that stochastic events such as landslides, hurricanes, and flash-flooding could obliterate the majority or all of the currently known plants with a single event. Approximately 20 mature plants and several immature plants have been observed in total. Plants that were observed ranged from seedlings to reproductively mature individuals. Seedlings are scarce, however, which suggests that the population may be declining.

The Hawai‘i DLNR, Division of Forestry and Wildlife (DOFAW) has largely funded this research by providing staff time to further investigate this species. The Hawai‘i Plant Extinction Prevention (PEP) Program focuses on conserving and restoring plants with less than 50 known wild individuals. Because *Cyanea konahuanuiensis* falls within that threshold, O‘ahu PEP is working closely with DOFAW staff to protect this critically rare taxon. The first goal of the PEP Program is to secure seeds or propagules from each individual mature plant for *ex situ* germplasm banking. The long-term goal for the PEP Program and DOFAW will be to collaborate in the effort to grow and out-plant nursery stock into appropriate restoration sites. Currently there are no protected (fenced) areas in similar habitat with comparable elevation, rainfall, humidity, and species composition on O‘ahu. Our recommendation is that additional fenced out-planting sites be established in appropriate areas of the Ko‘olau Mountains in order to establish multiple populations of this species. The authors emphasize the importance of prioritizing staff time to carry out further vegetation surveys in areas that have not been explored in recent history as this exciting new find shows that even seemingly well botanized areas in Hawai‘i may yet yield new discoveries.

In October 2013, immature fruits from two plants were collected and have since germinated at the UH Harold L. Lyon Arboretum after being directly sown on an agar medium. This is valuable information since at this time all observed fruits seem to be aborting prior to maturity. We recommend collecting immature fruit (or mature fruit if possible) from all reproductive individuals during future fruiting seasons in order to secure genetic representation from all reproductively mature individuals in *ex situ* collections.

The use of cellular phone-connected game cameras is recommended for monitoring of rare plants in remote locations. This novel use of game camera technology saved time and resources, optimizing the timing of visits and increasing the likelihood of making successful and representative observations required for the species description and fruit collections. This rapidly improving technology could have many positive impacts on monitoring rare plants for flower development, fruit development, herbivory impacts, and the effects of various seasonal events.

#### Specimens examined.

USA. Hawaiian Islands O‘ahu [East O‘ahu]: Paratypes: dried herbarium specimen BISH 1049144; and spirit collections: BISH 1059013, BISH 1059014, and BISH 1059015.

#### Discussion.

For over a century the taxa that currently comprise the genus *Cyanea* were recognized as two separate genera, *Cyanea* and *Rollandia*. The genus *Rollandia* was distinguished from *Cyanea* based on the single character of staminal column adnation to corolla in the former (Gaudichaud-Beaupré 1844; Hillebrand 1888; Lammers 1990; Rock 1919). Phylogenetic investigations of molecular data revealed that the taxa of *Rollandia* are embedded within the genus *Cyanea* (Lammers, 1993). Together with two species of *Cyanea* (*Cyanea acuminata* and *Cyanea grimesiana*), they form a clade referred to as the “*acuminata* clade” (Givnish et al. 1995). *Cyanea konahuanuiensis* most likely belongs in this clade based on the staminal column being adnate to the corolla tube. It is noteworthy to mention that *Cyanea konahuanuiensis* shares close geographic proximity to several taxa of the *acuminata* clade. Eight out of the nine previously recognized *Rollandia* taxa from this clade are endemic to O‘ahu (the ninth taxon, *Cyanea parvifolia* C. N. Forbes is only known from the type specimen collected on Kaua‘i) and six out of those eight are even more restricted, occurring only in the Ko‘olau Range. Of those six, one taxon (*Cyanea humboldtiana*) is known to occur on the summit ridges near *Cyanea konahuanuiensis*.

### Key to species

There is currently no published taxonomic key for *Cyanea* that includes the merger of the *Rollandia* clade. The following couplets can be inserted into the most recent revision of *Rollandia* (Lammers 1990 in Wagner et al. 1990) to separate *Cyanea konahuanuiensis* from *Cyanea humboldtiana*.

**Table:** 

2(1)	Inflorescence pendent, peduncles 50–250 mm long	**2’**
2’(2)	Upper leaf surface glabrous, calyx lobes 4–10 mm long	***Cyanea humboldtiana***
2’	Upper leaf surface densely pubescent, calyx lobes 16–18 mm long	***Cyanea konahuanuiensis***

### Conservation status

Using the IUCN Red List criteria (IUCN 2012), *Cyanea konahuanuiensis* falls into the Critically Endangered (CR) category, a rank given to species facing the highest threat of extinction in the wild, fitting the following criteria defined by IUCN Red List: B1) Extent of occurrence estimated to be < 100 km^2^ and D) number of mature individuals < 50. This species is currently known from only one population in two sub-gulches of a single stream drainage. It is our recommendation that *Cyanea konahuanuiensis* be evaluated by the United States Fish and Wildlife Service to be added as a Candidate for listing as Endangered under the Endangered Species Act of 1973. A recovery plan should be written and implemented.

## Supplementary Material

XML Treatment for Cyanea konahuanuiensis
